# A Review of the Mechanisms, Biological Basis, and Active Ingredients of Traditional Chinese Medicine Formulations in Regulating Organ Function to Alleviate Osteoporosis

**DOI:** 10.2174/0118715303447543260514041321

**Published:** 2026-06-29

**Authors:** Caihui Liu, Xingqin Wei, Houran Cao, Zhao Chen

**Affiliations:** 1 The Fifth College of Clinic Medicine, Guangzhou University of Chinese Medicine, 60 Hengfu Rd., Guangzhou, 510095, China;; 2 Guangdong Provincial Key Laboratory of Research and Development in Traditional Chinese Medicine, 60 Hengfu Rd., Guangzhou, 510095, China;; 3 The Second College of Clinic Medicine, Guangzhou University of Chinese Medicine, 111 Dade Rd., Guangzhou, 510120, China

**Keywords:** Osteoporosis, traditional Chinese medicine, endocrinium, mechanism, osteoblasts, osteoclasts

## Abstract

The occurrence and development of osteoporosis (OP) are closely related to the activity and metabolism of osteoblasts (OB) and osteoclasts (OC), which are significantly influenced by endocrine levels in the body. Traditional Chinese medicine (TCM) explains these processes through the theory of organ interconnection, suggesting that regulating the functions of the liver, spleen, and kidneys can influence hormone secretion, thereby alleviating OP symptoms. This concept forms the theoretical basis for the use of herbal medicines in OP treatment. In brief, the mechanism of action of the active components of TCM starts with their absorption through oral administration. Then, they act on metabolic organs and regulate multiple related target points, thereby influencing the synthesis and metabolic pathways of hormones and/or other endogenous substances associated with OP. Subsequently, they alter the activity of OB and OC as well as their autophagy processes. In this review, we summarize and evaluate the oral TCM used to alleviate OP, their mechanism of action, and pharmacodynamic research. We also discuss the biological associations between organ function and the effects of TCM on endocrine processes (hormones and metabolites). We evaluate the scientific validity, efficacy, advantages, and unique features of the organ-regulating treatment of OP by TCM.

## 1 INTRODUCTION

Osteoporosis (OP) is a chronic, potentially life-threatening condition. OP significantly affects the activity and quality of life of humans. Its prevalence and impact have expanded globally, with the affected population being increasingly younger [1].

Research has indicated that OP patients experience mobility issues and also carry a higher risk of falls, strokes, and muscle injuries than the general population. Studies on the pathogenesis and endocrine changes associated with the disease have shown that OP is a disorder caused by abnormalities in the metabolic system. Changes in the metabolism of hormones and substances lead to the weakening and destruction of bone structure, ultimately resulting in organic lesions [2]. This process manifests in bone tissue as a disruption of the balance between the formation and resorption of bone. This disruption of balance results in bone destruction/disintegration, with regeneration and remodeling slowing down (or even ceasing) [2, 3]. In-depth studies have revealed that this disruption of balance is manifested at the cellular level by an imbalance between osteoblasts (OB) and osteoclasts (OC) in terms of activity and numbers. That is, apoptosis is increased, metabolic function in OB is weakened, and OC exhibits increased activity and abnormal proliferation [4, 5]. These cellular-level changes are closely related to mitochondrial autophagy within OB. Under the influence of hormonal and endocrine changes, mitochondrial autophagy and oxidative stress in OB are exacerbated, severely impairing bone regeneration and the reconstruction of damaged internal structures. Concurrently, OC undergoes abnormal proliferation due to the same factors, thereby exacerbating the destruction of internal bone tissue [6, 7]. Therefore, the key to treating OP lies in restoring the balance between OB and OC, promoting the regeneration/reconstruction of internal bone structure to return to homeostasis, and, ultimately, forming stable and robust bone tissue [8, 9].

The primary treatment approaches for OP are regulating hormonal balance and restoring cellular balance. The former primarily targets the underlying causes of OP, specifically imbalances in the levels and metabolism of endogenous substances (*e.g.*, estrogen) within the body. This strategy is achieved through pharmacological intervention to adjust hormone levels to normal ranges [10, 11]. This method has a long history of clinical application. However, it cannot address functional issues in organs and OB, so it alleviates only symptoms. In addition, prolonged use of hormonal medications at high doses may lead to severe side effects and dose dependency, affecting health and quality of life. Therefore, careful consideration of the benefits and risks is necessary when employing this approach [12, 13].

Stem cell therapy has emerged as a research and clinical “hotspot” in recent years. The introduction of stem cell technology can partially restore the balance between OC and OB, enabling a degree of self-regulation. Compared with hormone therapy, it has fewer side effects and offers relatively sustained and stable efficacy. However, stem cell therapy has several unknown aspects, and its risks and side effects require further investigation. In addition, this method involves regulating the internal environmental homeostasis related to OP through intervention with exogenous substances, with limited impact on its endogenous dynamics (*i.e.*, endocrine and metabolic regulatory functions in organs). There is also the issue of recurrence after discontinuing treatment [14-16].

All these methods have certain limitations, so seeking new treatment strategies has become an important direction for the prevention and treatment of osteoporosis (OP). Traditional Chinese medicine (TCM), as an important part of China's excellent traditional culture, has a history of thousands of years in China. TCM treatment of diseases usually has the characteristics of multiple components and multiple targets, with relatively mild effects and fewer side effects [17-19]. With the deepening of modern research, the mechanism of TCM in treating osteoporosis has expanded from a single pathway to a systematic network regulation, such as through the “gut-bone” axis and other pathways [20]. In addition, the integration and exploration of TCM with modern technologies such as network pharmacology, multi-omics, and nanomedicine have shown broad new prospects for the treatment of osteoporosis [18, 21].

In the theoretical system of TCM, osteoporosis can be classified as “bone atrophy”, “bone depletion”, and “bone arthralgia”, with the concept derived from the ancient TCM classic text, the Huangdi Neijing [22]. The main causes and pathogenesis are attributed to kidney essence deficiency, spleen dysfunction, liver blood insufficiency, and blood stasis blocking the meridians [23]. In modern medicine, the mechanism of action of oral traditional Chinese medicine differs from that of drug supplementation or stem cell therapy. The most distinctive feature of oral TCM lies in its ability to act on the liver, spleen, and kidneys after oral absorption, thereby altering the endocrine functions and levels of these organs. Through this process, oral TCM also influences the activity of OB/OC and affects cellular processes such as mitochondrial autophagy, oxidative stress, and ferroptosis [24-26].

Identifying the primary regulatory mechanisms underlying OP progression is essential for developing and optimizing treatment strategies. Research has indicated that OP, along with the OB/OC balance, mitochondrial autophagy in relevant cells, oxidative stress, and other physiological processes, is closely associated with the function, morphology, and expression of multiple target points and signal transduction pathways in organs [27-29]. This theory shares common ground with the principles of diagnosis and treatment in TCM. TCM emphasizes regulating the functions of the kidneys, liver, and spleen to restore their normal functions and activities, thereby maintaining the OB/OC balance, which exhibits strong sustainability. The scientific basis for this theory can be explained through the regulatory mechanisms of the OB/OC balance. Research has indicated that multiple targets related to OB and OC are regulated by upstream targets widely distributed in the kidneys, liver, and spleen, and that the metabolic mechanisms involved are closely related to the functions of these organs [30-32]. When the active components of oral medications are absorbed into the bloodstream, they first pass through these organs. This approach may result in metabolic losses and a slower onset of action compared with direct injection into the affected area. As the functions of the organs involved in OP regulation are restored, their related metabolic processes return to normal, thereby enhancing the body's self-regulatory mechanisms. This information provides a material basis for the more stable and sustained effects of oral TCM [33-35].

## 2 MATERIALS AND METHODS

### 2.1 Search Strategy

A comprehensive search was conducted across multiple electronic databases, including PubMed and Web of Science for English literature, as well as CNKI and WanFang for Chinese literature, from their inception until August 1, 2025. The search terms were constructed around core concepts such as “osteoporosis,” “Traditional Chinese Medicine,” “Kidney-tonifying,” “Spleen-invigorating,” and combined with keywords like “mechanism,” “signal pathway,” and “active ingredients” using Boolean operators.

### 2.2 Study Selection

The study selection process was carried out by Caihui Liu and Xingqin Wei based on pre-defined eligibility criteria. The population of interest included *in vivo* osteoporotic animal models, relevant cell lines, or human patients. Interventions involved TCM formulas, single herbs, or extracts explicitly linked to TCM theories of regulating “Kidney,” “Spleen,” or “Liver” function. Studies required a control group and had to report outcomes related to bone density, biomechanics, or associated molecular mechanisms. The selection process involved an initial screening of titles and abstracts, followed by a full-text assessment of potentially eligible studies. Any disagreements between reviewers were resolved through discussion or consultation with Houran Cao, and reasons for exclusion at the full-text stage were documented.

### 2.3 Data Extraction and Synthesis

Key data were extracted from each included study, encompassing study characteristics, details of the TCM intervention, the associated traditional medical theory, reported bioactive constituents, and all relevant pharmacodynamic and mechanistic outcomes. Firstly, the studies were initially categorized based on the core organ systems targeted by the intervention, such as the renal system, splenic system, and hepatic system. Subsequently, we systematically organized and summarized the biological mechanisms involved within each category. These mechanisms include the regulation of signaling pathways related to OB/OC balance and the modulation of oxidative stress and inflammation levels, thereby clearly presenting the overall framework, main findings, and internal connections of current research.

## 3 THE ENDOCRINE REGULATORY MECHANISM OF OP

Endocrine research has focused on the tissue distribution, metabolic mechanisms, functions, and roles of specific hormones or related metabolites. Some studies have begun to explore OP-related endocrine mechanisms. Multiple endogenous substances influencing OP (particularly hormones) originate from organs described in classical TCM theory as having significant effects on bones: the kidneys, spleen, and liver. Therefore, research on the endocrine-level mechanisms of OP progression and key biomarkers can link bones with these organs, thereby elucidating the scientific basis of the diagnostic theories and treatments of TCM and identifying better entry points for their optimization and application [36, 37]. An integrated schematic overview of these pathological mechanisms from both TCM and Western medicine perspectives is presented in Fig. (**1**).

However, the internal use of TCM primarily targets OP-related organs, restoring organ and bodily functions to regulate endocrine levels back to normal states, resulting in relatively more stable and sustained therapeutic effects. This strategy aligns with the syndrome differentiation and treatment theory of TCM for OP, which states that the “kidney stores essence and governs bones,” the “liver governs the circulation and connects to the tendons,” and the “spleen governs transformation and is responsible for muscles” [38-40]. Recent studies on the pharmacological mechanisms of OP have shown that bones (as endocrine organs) interact with and influence multiple organs, thereby providing scientific validation of the aforementioned TCM theories from a physiological perspective. Therefore, TCMs that regulate organ functions, promote qi and blood circulation, strengthen bones, and alleviate arthralgia have garnered increasing attention in recent years [41-43].

### 3.1 Estrogen Pathway

Under the theoretical framework of traditional Chinese medicine that “the kidney stores essence and governs reproduction and bone metabolism”, the functional state of the kidney is closely related to the regulation of estrogen by the hypothalamus-pituitary-gonadal axis. The prosperity or decline of “kidney essence” can reflect and regulate the function of this axis, thereby influencing estrogen levels and thus affecting the balance of bone metabolism [44, 45]. Metabolomics analysis based on animal models further reveals the chemical nature of this association, demonstrating that in the absence of estrogen (such as ovarian removal), the levels of key metabolites such as creatine, β-hydroxybutyric acid, and urea in kidney tissues undergo significant changes, suggesting that the energy metabolism and acid-base balance functions of the kidneys are impaired [45]. This metabolic disorder not only reflects the modern connotation of kidney deficiency but may also interfere with the normal transduction of the estrogen signaling pathway by affecting the calcium and phosphorus homeostasis throughout the body and the redox state of the bone microenvironment. Estrogen is a key hormone that maintains the balance of bone metabolism. Its signals are mainly transmitted by regulating the downstream RANKL/OPG and Wnt/β-catenin two core pathways [46]. However, the absence of estrogen inactivates these two core pathways, leading to bone resorption and imbalance in bone metabolism.

### 3.2 Vitamin D and the Parathyroid Hormone Pathway

The traditional Chinese medicine theory that “the kidney governs bones and produces marrow” corresponds in modern medicine to chronic kidney disease and mineral and bone metabolism disorder (CKD-MBD). The core mechanism of this syndrome lies in the fact that renal failure leads to the imbalance of two key physiological processes: Firstly, the loss of renal 1α-hydroxylase function, which is unable to convert 25-(OH)D_3_ into the active form 1,25-(OH)_2_D_3_ (calcitriol), resulting in an absolute deficiency of active vitamin D synthesis and a lack of bone mineralization raw materials; Secondly, the renal disorder in phosphorus excretion triggers secondary hyperparathyroidism, with abnormal elevation of parathyroid hormone (PTH), driving abnormal active bone resorption [47, 48].

The deficiency of active vitamin D leads to reduced calcium absorption in the intestine, subsequently causing secondary hyperparathyroidism (increased PTH). The elevated PTH acts on OB, thereby promoting the upregulation of RANKL and the downregulation of OPG expression, increasing the RANKL/OPG ratio, which excessively activates OC and drives bone resorption [49]. Metabolomics studies further reveal that in the serum of CKD patients with elevated PTH, the levels of hydroxyproline, dopamine metabolites, and metabolites of the tryptophan/serotonin pathway are significantly altered. These metabolic disturbances are directly related to abnormal protein synthesis, energy metabolism, and steroid hormone metabolism, jointly exacerbating the imbalance of bone turnover and the destruction of bone microstructure [50].

### 3.3 Growth Hormone/Insulin-Like Growth Factor-1 Pathway

Under the theoretical framework of traditional Chinese medicine that “the liver governs the regulation of qi and is associated with the tendons in the body”, the functional state of the liver is closely related to the regulation of the growth hormone-insulin-like growth factor-1 axis (GH/IGF-1) on the overall anabolic process throughout the body. Experimental studies using a liver-specific gene knockout mouse model have shown that the absence of liver-specific growth hormone receptors can directly lead to severe low IGF-1 levels, significant cortical bone loss, and a decline in mechanical properties [51]. Therefore, “liver qi” can reflect and regulate the function of this axis, thereby maintaining normal levels of IGF-1 synthesis and secretion, and maintaining the stability of bone metabolism and structure through the functional synergy between muscles and bones. When the GH/IGF-1 axis is dysfunctional, a “resistance” state may occur, where serum IGF-1 levels increase, but OB responsiveness decreases, while OC activity is relatively enhanced, resulting in insufficient bone formation and relatively active bone resorption, ultimately leading to bone metabolism imbalance and bone loss [52].

### 3.4 Liver and Immunity

The endocrine regulation of osteoporosis is not isolated; its effects are greatly influenced by immune homeostasis. According to traditional Chinese medicine, “the spleen governs digestion and transformation”, and when the spleen is deficient, the transformation function is disrupted, leading to “internal generation of dampness and turbidity”, which is similar to the chronic low-grade inflammation in modern medicine [53]. In this inflammatory state, the immune system is overactivated and releases a large amount of pro-inflammatory cytokines such as Tumour necrosis factor-alpha (TNF-α) and Interleukin-1 beta (IL-1β), which can strongly drive the differentiation of OC and cause “inflammatory bone loss”. Modern research provides direct evidence for this: in the inflammatory osteoporosis mouse model induced by LPS, the immune homeostasis of the spleen, as a key immune organ, is disrupted. By intervening to upregulate the expression of anti-inflammatory and anti-osteoclastogenic factors nterferon-gamma (IFN-γ)and IL-10 in the spleen, it can effectively inhibit pro-inflammatory factors and alleviate bone loss [54]. This confirms that the balance of the “spleen-immune axis” is a key link in maintaining bone homeostasis, and also provides a molecular basis for the strategy of “strengthening the spleen and transforming the dampness” in traditional Chinese medicine to regulate the immune system and prevent osteoporosis.

## 4 THE MAIN PATHWAYS AND REGULATORY MECHANISMS OF THE THERAPEUTIC TARGETS OF OP

As mentioned previously, the occurrence of osteoporosis is closely related to the endocrine disorders caused by the dysfunction of organs such as the kidneys, liver, and spleen. However, these diverse systemic signals do not directly act on the bones but converge on a limited number of key cell pathways within the bone tissue. Through precise regulation of the fates of OB and OC, they ultimately lead to the imbalance of bone homeostasis. This work will thoroughly analyze these core execution pathways and molecular targets and clarify the specific cellular and molecular mechanisms by which systemic factors lead to local bone loss.

### 4.1 RANKL/RANK/OPG Pathway

The literature indicates that the formation of bone structure *in vivo* is regulated by OB, which drives the occurrence and development of OP. OC significantly promotes bone resorption. The balance between OB and OC is the primary regulatory mechanism for bone regeneration [55-57]. One of the core switches of this balance is the RANKL/RANK/OPG system. Both *in vitro* and *in vivo* studies have confirmed that OB and OC are regulated by multiple proteins, such as Macrophage Colony-Stimulating Factor (M-CSF) and RANKL. Their function is to bind to receptors like C-FMS and RANK, activating the receptor-related pathways and promoting OC differentiation [58-62]. Specifically, the precursor cells of OB and OC express RANK on their surface. RANKL is the ligand of the receptor RANK, and its specific binding induces the differentiation of OC, thereby mediating bone resorption. OPG is a soluble RANKL decoy receptor, mainly produced by OB, which can bind to RANKL and prevent its binding to RANK, thereby inhibiting the differentiation of OC [63]. This pathway is a convergence point for multiple upstream signals: as mentioned previously, estrogen deficiency, elevated PTH, *etc.*, affect this pathway by regulating the expression ratio of RANKL and OPG in OB, thereby influencing the strength of bone resorption (Fig. **2**).

### 4.2 Wnt/β-Catenin Signaling Pathway

As mentioned in the previous section, in bone, OB plays a dominant role in bone formation, and activation of the Wnt signaling pathway can enhance the differentiation of Bone Marrow Mesenchymal Stem Cells (BMSCs) towards OB, thereby promoting bone regeneration [64]. In the classical pathway, when the Wnt signaling pathway is activated, the Wnt ligands bind to the curled proteins on the cell membrane and the Low-density lipoprotein receptor-related protein 5/6 receptors, resulting in the inhibition of the intracellular β-catenin degradation complex (such as Glycogen synthase kinase-3 beta), causing an increase in the concentration of β-catenin in the cytoplasm and its transport into the nucleus. In the nucleus, β-catenin acts as a transcriptional co-activator, initiating the expression of key osteogenic genes such as Runx2 and osteocalcin, ultimately promoting bone formation [65]. This process is strictly controlled by endogenous inhibitory proteins, among which sclerostin and Dickkopf-1 are the two most important physiological antagonists, which inhibit this pathway by blocking the signal receptors [66]. In the pathological state of osteoporosis, such as estrogen deficiency, inhibiting this pathway leads to decreased OB activity and differentiation, resulting in insufficient bone formation and, subsequently, bone loss (Fig. **2**).

### 4.3 The Influence of Autophagy on the Balance Between OB and OC and Its Regulatory Mechanism

The effects of cellular autophagy on OP and its associated OB and OC are not isolated phenomena. This process intersects with the aforementioned metabolic pathways, hormone secretion, and inflammatory responses in numerous ways. He *et al.* reported that cellular autophagy, a key factor in exacerbating OP, acts through changes in the body's energy supply mediated by RANKL [67]. Another study suggested that if the intracellular Bone Morphogenetic Protein(BMP) levels of Activin receptor-like kinase 3 (Bone morphogenetic protein receptor type 1A) are below normal, it can affect cellular autophagy and exacerbate bone loss, indicating that cellular autophagy is influenced by the expression of proteins [68]. Inflammation is also a frequently studied area in research on apoptosis. Studies have shown that multiple inflammatory factors influence cellular autophagy significantly, such as Interleukins (ILs) and Tumour Necrosis Factor-alpha (TNF-α). Under the influence of related regulatory pathways or key regulatory factors (*e.g.*, Nuclear factor kappa-B[NF-κB]), these factors accumulate in specific locations or are metabolically produced in large quantities, thereby affecting the key regulatory pathways of cellular autophagy, accelerating the latter, and, ultimately, inducing or exacerbating OP [69, 70]. Metabolic diseases (*e.g.*, diabetes mellitus and hyperlipidemia) are also important factors in inducing or exacerbating OP. The metabolic disorders of glucose and lipids caused by these conditions exacerbate inflammatory responses in the liver and kidneys. Such exacerbation affects organ function and accelerates mitochondrial oxidative stress due to the accumulation of lipid and glucose metabolic products, thereby affecting the activity of Protein Kinase B (PKB), Mechanistic target of rapamycin, and others, and ultimately accelerating cellular autophagy [71, 72]. Ferroptosis can be exacerbated by lipid metabolism disorders and abnormal levels of lipid oxidation, which also induce mitochondrial oxidative stress and affect the AMP-activated protein kinase pathway, ultimately leading to cellular autophagy, apoptosis, and exacerbation of OP [25].

Hence, the regulation of OP by cellular autophagy is the result of multifactorial and multi-translational mechanisms, with these factors typically coexisting. The primary distinction lies in which factors serve as the dominant driver. Overall, three main conclusions can be drawn. Firstly, cellular autophagy in bone tissue, its internal environment, and associated organs inevitably leads to OP, making the regulation of cellular autophagy a critical target for drug-based treatment of OP [73, 74]. Secondly, the regulatory pathways and multiple regulatory sites of autophagy are distributed across the liver, kidneys, and spleen. The diagnosis and theory of TCM in OP treatment protocols primarily target the regulation of these organs. Therefore, conducting screening and evaluation of herbal medicines and their active components based on relevant targets is rational and has scientific merit [75, 76]. Thirdly, the interplay between OP and other metabolic disorders, such as diabetes mellitus and hyperlipidemia, should not be overlooked. Metabolic diseases and their associated inflammatory responses produce many endogenous substances that induce autophagy and apoptosis. Hence, drug regimens should not be limited to targeting the site of OP occurrence. This approach aligns with the focus of TCM on regulating bodily balance and restoring organ function, further highlighting the distinctive features and advantages of oral TCM [77, 78].

## 5 RESEARCH ON THE MECHANISM OF TCM IN REGULATING ORGANS FOR THE TREATMENT OF OP

The systemic causes of osteoporosis originate from the dysfunction of organs such as the kidneys, liver, and spleen, which leads to endocrine and immune disorders [79]. These disordered signals from different physiological systems eventually converge through a common downstream pathway to the bone tissue, disrupting the delicate balance between bone formation and bone resorption, thereby causing bone loss [80]. The core concept of traditional Chinese medicine in treating osteoporosis lies in “treating according to the syndrome, regulating the internal organs”. This work intervenes in the above regulatory network through drugs under different therapeutic principles, such as tonifying the kidneys, tonifying the liver, and tonifying the spleen, in multiple links and multiple targets, to systematically restore bone homeostasis. Based on modern pharmacological research, various traditional Chinese medicines exert their therapeutic effects by acting on the core endocrine axes and cell signaling pathways described earlier. The mechanisms by which traditional Chinese medicine regulates these visceral functions to treat osteoporosis are detailed in Table **1**.

### 5.1 Mechanistic Study of TCM for Kidney Regulation in OP Treatment

The traditional Chinese medicine for tonifying the kidney acts on the core pathways of bone metabolism through multiple targets. *In vitro* cellular experiments have confirmed that *Epimedium brevicornum*, the active ingredient icariin, by activating the Wnt/β-catenin signaling pathway, promotes OB differentiation to enhance bone formation. This has been confirmed in animal models, where it simultaneously inhibits the generation of OC and bone resorption by suppressing the downstream signaling pathways mediated by RANKL, thereby regulating the bone metabolism homeostasis in a bidirectional manner and alleviating osteoporosis [81, 82]. Animal studies have confirmed that psoralen, an active component of *Psoralea corylifolia L.*, acts as a phytoestrogen. By selectively activating the ERα receptor, it upregulates BMP-2 expression to promote bone formation and regulates the OPG/RANKL balance to inhibit osteoclastic resorption, thereby systematically improving osteoporosis and fracture healing. [83]. Both *in vivo* and *in vitro* studies have confirmed that the total flavonoids of *Drynaria fortunei* activate the Wnt/β-catenin and BMP-Smad pathways, upregulate key transcription factors such as Runx2 and Osterix, and regulate the RANK/RANKL/OPG pathway, downregulate the expression of RANK receptors, jointly promoting osteogenesis and inhibiting osteoclastogenesis, thereby treating osteoporosis [84, 85]. In summary, traditional Chinese medicine, by tonifying the kidney and replenishing essence, can not only improve kidney function but also act on the bones, achieving the effect of treating both the symptoms and the root cause.

### 5.2 Mechanistic Study of TCM for Liver Regulation in OP Treatment

Based on the theory that “the liver governs excretion and is associated with tendons”, the herbs for tonifying the liver are mainly related to the regulation of the GH/IGF-1 axis. The dysfunction of this axis affects the coordination of muscles and bones and the anabolic process. At the same time, the liver, losing its excretory function, is prone to oxidative stress and exacerbates bone cell damage [86].

The herbs for tonifying the liver affect bone homeostasis by promoting anabolic metabolism and antioxidant pathways. It has been confirmed in animal models that *Eucommia ulmoides Oliv.* extract mainly inhibits oxidative stress by activating the Nuclear factor erythroid 2–related factor 2/Heme oxygenase-1 signaling pathway, serving as an upstream core therapeutic target. This pathway regulates the intestinal-kidney calcium transport channels and the coupling balance of OB and OC downstream, thereby maintaining calcium homeostasis and improving bone metabolism [87]. In preclinical animal models, *Bupleurum chinense DC.* directly targets and inhibits liver oxidative stress and inflammatory signals through its active component, Saikosaponin A. It blocks the abnormal signal transmission of liver-derived inflammatory factors (such as ROS and IL-17) to the bones. At the same time, Saikosaponin A induces OC ferroptosis by inhibiting the Nuclear factor erythroid 2-related factor 2/Solute carrier family 7 member 11/Glutathione peroxidase 4 pathway, acting both on inter-organ signals and directly on bone cells, causing OC death [86, 88]. Based on the theory that “the liver governs tendons”, traditional Chinese medicine provides a unique perspective for the treatment of osteoporosis by regulating the liver through syndrome differentiation and combining the multi-component and multi-pathway effects of Chinese herbs, demonstrating the advantages of the holistic view and syndrome differentiation and treatment of traditional Chinese medicine.

### 5.3 Mechanistic Study of TCM-based Regulation of the Spleen in OP Treatment

The chronic low-grade inflammatory state corresponding to spleen deficiency is a key upstream factor that disrupts bone metabolic homeostasis and accelerates bone loss. Therefore, tonifying spleen-based traditional Chinese medicines mainly intervene in the process of osteoporosis from the aspects of immune regulation and cell fate control. Animal studies have confirmed that the astragalus polysaccharides in *Astragalus membranaceus* can significantly inhibit the production of pro-inflammatory factors (such as TNF-α, IL-6), which can promote the expression of RANKL. Thus, astragalus, through its systemic anti-inflammatory effect, inhibits the abnormal activation of the RANKL/RANK pathway at the upstream level, thereby suppressing the generation of OC [89, 90]. Animal studies have confirmed that the codonopsis polysaccharide in *Codonopsis pilosula* reduces bone loss in OVX rats. *In vitro* cellular experiments further indicate that it significantly activates the Wnt/β-catenin signaling pathway, thereby upregulating the expression of the key osteogenic transcription factor RUNX2, promoting the differentiation of bone marrow mesenchymal stem cells into OB [91]. These studies reflect the holistic view of “strengthening the spleen and muscles to strengthen the bones” in traditional Chinese medicine. Additionally, modern research reveals its scientific connotations at the molecular level, providing a theoretical basis and innovative ideas for the prevention and treatment of osteoporosis through the combination of traditional Chinese and Western medicine.

## CONCLUSION

This article mainly focuses on preclinical research on osteoporosis. Subsequent studies will involve clinical research and literature review.

OP, a common metabolic bone disease, primarily results from an imbalance in the functions of OB and OC [92]. Western medical treatments mainly involve hormone-based medications and bisphosphonates, which inhibit bone resorption. However, long-term use may lead to gastrointestinal discomfort and osteonecrosis of the jaw, and it also carries cardiovascular risks [93]. These adverse effects of Western medical treatments have led to growing interest in TCM for OP treatment. According to TCM theory, “the kidneys govern bone marrow production,” attributing OP to “kidney essence deficiency.” It also emphasizes the important roles of “the spleen governing transformation and transportation” and “the liver governing the free flow of qi” in bone metabolism [94]. This holistic approach guides treatment strategies that regulate bone metabolic balance through multi-targeted, multi-pathway synergistic effects, offering symptomatic and root-cause treatment. However, research on TCM-based treatment for OP faces numerous challenges. To fully integrate TCM into modern prevention and treatment systems for OP, several challenges must be addressed.

The primary issue is the standardization and internationalization of clinical evidence. Most clinical studies using TCM suffer from small sample sizes, short follow-up periods, and inconsistent evaluation criteria, making it difficult to gain recognition from the international medical community. Another challenge is elucidating the mechanism of action of TCM components. Recent studies have shown that TCM can exert its effects through multiple signaling pathways, such as Wnt/β-catenin and OPG/RANKL/RANK; however, these studies remain fragmented and lack systematic coherence [95]. Another challenge is the safety of TCM, particularly for those containing heavy metals or toxic components, which necessitates the establishment of more comprehensive quality control standards [96]. The development of TCM in the prevention and treatment of OP requires a multi-pronged strategy. The core task in advancing the clinical translation of TCM for OP lies in addressing the challenges of herbal formulation standardization, quality control, and clinical validation. In basic research, emerging technologies such as systems biology and network pharmacology should be employed to elucidate the mechanism of action of TCM components. In clinical research, large-scale, multicenter, randomized controlled trials should be conducted according to international standards. In drug development, pharmaceutical technologies can be leveraged to optimize and improve TCM [97-100]. In addition, integrated diagnostic and treatment guidelines for TCM and Western medicine should be established to leverage the strengths of both systems and provide patients with superior treatment options. Notably, TCM possesses unique advantages in the prevention of OP. Through constitutional identification and early intervention, TCM can achieve the goal of “preventing disease before it occurs,” which is important in reducing OP incidence. With the advancement of the “Healthy China” strategy and the acceleration of TCM's modernization, TCM could play an increasingly important role in global prevention and treatment of OP, providing solutions to address the health challenges posed by an aging society. Achieving this vision requires directly confronting and overcoming the core challenges of herbal standardization, quality controllability, and large-scale clinical evidence.

## Figures and Tables

**Fig. (1):**
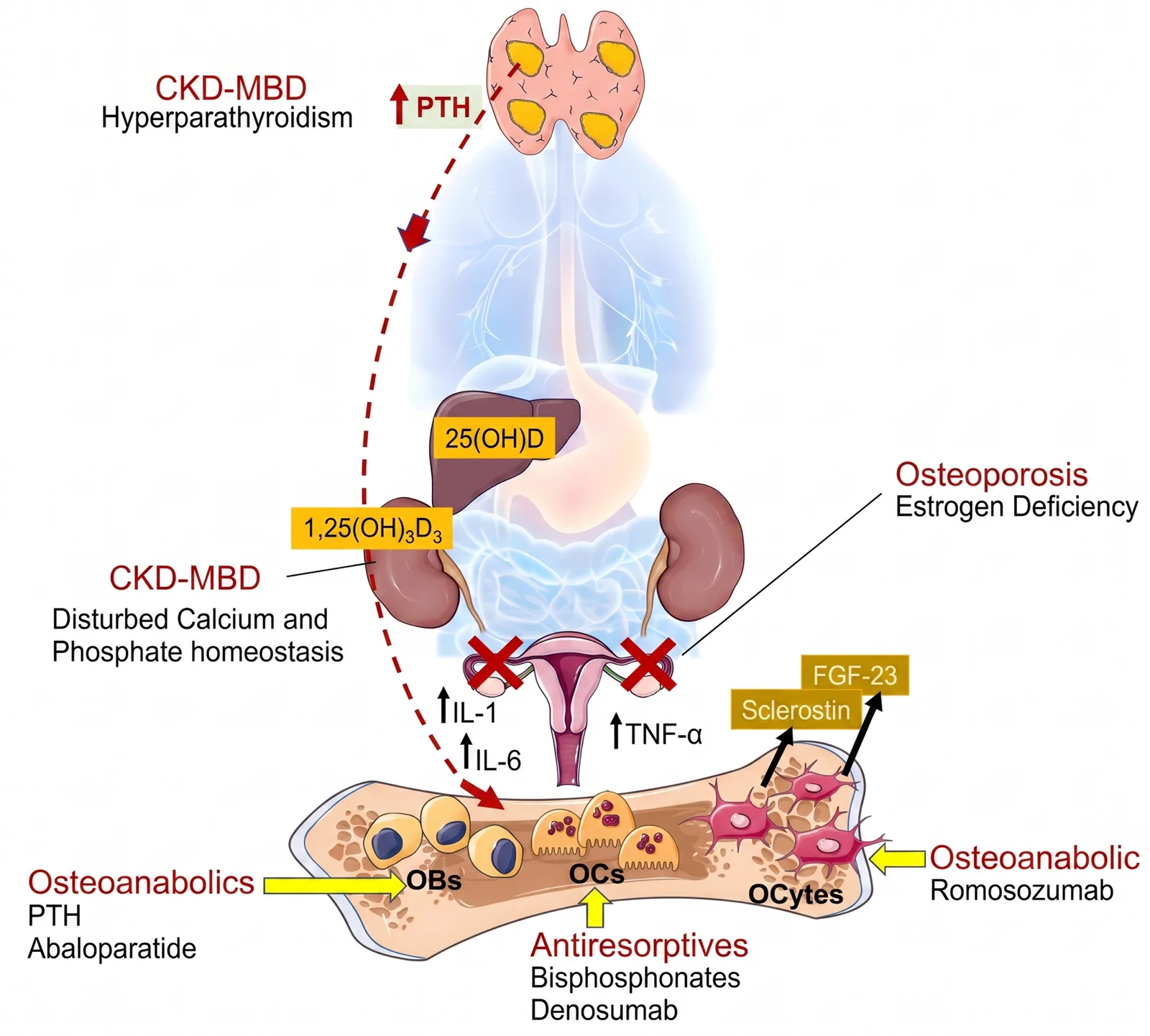
Integrated Schematic Diagram of the Pathological Mechanisms of Osteoporosis in Traditional Chinese Medicine and Western Medicine. Literature Source: Osteoporosis and Chronic Kidney Disease-Mineral and Bone Disorder (CKD-MBD): Back to Basics. Am J Kidney Dis. 2021;78(4):582-589.

**Fig. (2):**
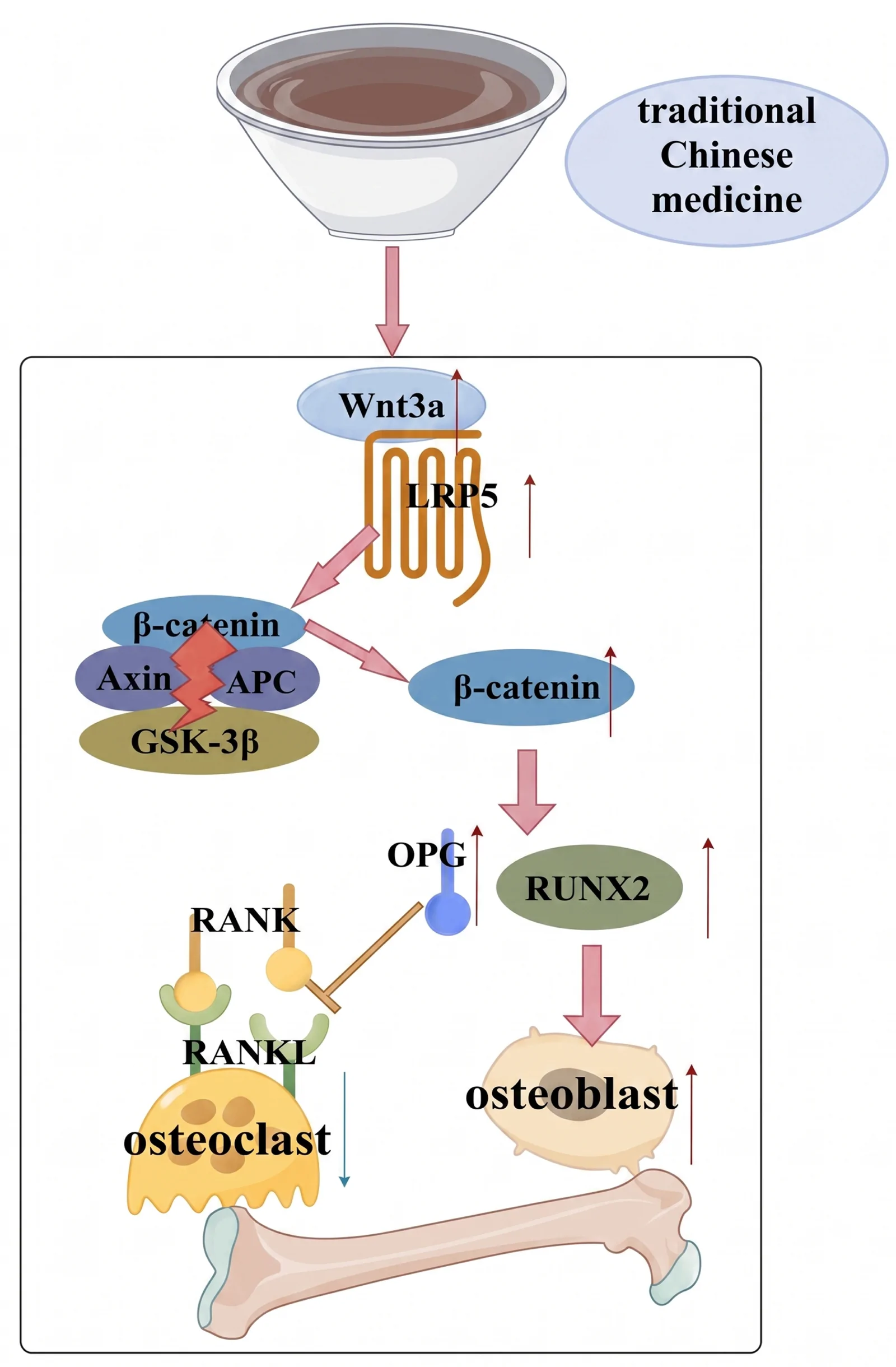
Dual Primary Pathway Mechanisms of Traditional Chinese Medicine in Treating Osteoporosis. (Literature Source: Hydrolyzed egg yolk peptide prevented osteoporosis by regulating Wnt/β-catenin signaling pathway in ovariectomized rats. Sci Rep. 2024;14(1):10227. Published 2024 May 3).

**Table 1:** Mechanisms of TCM in treating OP by regulating visceral functions.

Viscera Regulation	Representative Herb	Key Active Constituent	Involved Core Signaling Pathway(s)/Target(s)	Refs.
Tonifying Kidney	*Epimedium brevicornum*	icariin	Wnt/β-catenin, RANKL pathway	[82, 83]
-	*Psoralea corylifolia L.*	Psoralen	ERα, BMP-2, OPG/RANKL	[84]
-	*Drynaria fortunei*	The total flavonoids of *Drynaria fortunei*	Wnt/β-catenin, BMP-Smad, RANK/RANKL/OPG, Runx2, OSX	[85, 86]
Tonifying Liver	*Eucommia ulmoides Oliv.*	extractive	Nrf2/HO-1 pathway	[88]
-	*Bupleurum chinense DC.*	Saikosaponin A	Oxidative stress/Inflammation, Nrf2/SLC7A11/GPX4	[87, 89]
Tonifying Spleen	*Astragalus membranaceus*	astragalus polysaccharides	Inflammatory signaling (TNF-α, IL-6), RANKL/RANK	[90, 91]
-	Codonopsis pilosula	codonopsis polysaccharide	Wnt/β-catenin pathway, RUNX2	[92]

## LIST OF ABBREVIATIONS

OP: Osteoporosis
OB: Osteoblasts
OC: Osteoclasts
TCM: Traditional Chinese Medicine
RANKL: Receptor Activator Of Nuclear Factor-kappaB (NF-κB) Ligand
OPG: Osteoprotegerin
Runx2: Runt-related Transcription Factor 2
CKD: Chronic Kidney Disease
PTH: Parathyroid Hormone
GH: Growth hormone
IGF-1: Insulin-like Growth Factor-1
IFN-γ: Interferon-gamma
BMP: Bone Morphogenetic Protein
IL: Interleukins
TNF-α: Tumour Necrosis Factor-alpha
M-CSF: Macrophage Colony-stimulating Factor
NF-κB: Nuclear Factor Kappa-B
